# Re-examination of the cranial osteology of the Arctic Alaskan hadrosaurine with implications for its taxonomic status

**DOI:** 10.1371/journal.pone.0232410

**Published:** 2020-05-06

**Authors:** Ryuji Takasaki, Anthony R. Fiorillo, Ronald S. Tykoski, Yoshitsugu Kobayashi

**Affiliations:** 1 Faculty of Biosphere-Geosphere Science, Okayama University of Science, Ridaicho, Kita-ku, Okayama city, Okayama, Japan; 2 Perot Museum of Nature and Science, Dallas, Texas, United States of America; 3 Hokkaido University Museum, Kita 10, Nishi 8, Kita-ku, Sapporo, Hokkaido, Japan; Universidade de Sao Paulo, BRAZIL

## Abstract

Hadrosaurid fossils from the Liscomb Bonebed (Prince Creek Formation, North Slope, Alaska) were the first dinosaur bones discovered from the Arctic. While the Prince Creek Formation hadrosaurids were long identified as *Edmontosaurus*, a member of the sub-clade Hadrosaurinae, they were recently assigned to a newly-erected taxon, *Ugrunaaluk kuukpikensis*. However, taxonomic status of the new taxon is ambiguous largely due to the immature nature of the specimens upon which it was based. Here we reexamine cranial elements of the Prince Creek Formation hadrosaurine in order to solve its taxonomic uncertainties. The Prince Creek Formation hadrosaurine possesses a short dorsolateral process of the laterosphenoid, one of the diagnostic characters of *Edmontosaurus*. The Prince Creek Formation hadrosaurine also shows affinity to *Edmontosaurus regalis* in the presence of a horizontal shelf of the jugal. Our morphological comparisons with other North American *Edmontosaurus* specimens and our phylogenetic analyses demonstrate that the Prince Creek Formation hadrosaurine should be re-assigned to *Edmontosaurus*. Because the Prince Creek Formation *Edmontosaurus* shows differences with lower latitude *Edmontosaurus* in a dorsoventrally short maxilla, presence of a secondary ridge on the dentary teeth, and the absence of the transverse ridge between basipterygoid processes of the basisphenoid, we consider that the Prince Creek Formation *Edmontosaurus* should be regarded as *Edmontosaurus* sp. until further discoveries of mature hadrosaurines from the Prince Creek Formation Bonebed and/or equivalently juvenile *Edmontosaurus* specimens from the lower latitudes allow direct comparisons. The retention of the Prince Creek Formation hadrosaurine as *Edmontosaurus* re-establishes a significant latitudinal distribution for this taxon. Despite the large latitudinal distribution of the taxon, the morphological disparity of *Edmontosaurus* is small within Hadrosaurinae. The small morphological disparity may be related to the relatively low latitudinal temperature gradient during the latest Cretaceous compared to present day, a gradient which might not have imposed significant pressure for much morphological adaptations across a broad latitudinal range.

## Introduction

Increasing work in the Late Cretaceous deposits of the palaeo- Arctic is revealing higher dinosaur diversity in the region than had been previously recognized [1–3]. The most abundant Arctic dinosaur fossil record comes from Alaska where multiple dinosaur groups (ankylosaurid [4], ceratopsids [5–7], dromaeosaurids [8], hadrosaurids [9, 10], ornithomimosaur [11], basal ornithopods [12, 13], pachycephalosaurid [14, 15], troodontids [8, 16, 17], and tyrannosaurids [8, 18–20]) are known from skeletal material, and numerous dinosaur footprints [1, 21–26].

Among the most abundant dinosaurs collected in Alaska are the bones attributable to members of the large herbivorous group, the Hadrosauridae. Almost all of the hadrosaurid body fossils in Alaska come from the Upper Cretaceous Prince Creek Formation (PCF), which crops out along the Colville River, North Slope, Alaska. The Liscomb Bonebed in the PCF is especially rich in hadrosaurid fossils (up to 220 elements/m^2^ [27]). The dominant Liscomb hadrosaurid taxon was initially considered to belong to the clade Lambeosaurinae [28], but Nelms [29] later identified the Liscomb hadrosaurid as the hadrosaurine, *Edmontosaurus saskachewanensis*, which is now a junior synonym of *Edmontosaurus annectens* [30]. Although subsequent work reached a general consensus that the Liscomb hadrosaurid, as well as hadrosaurid materials from other Prince Creek Formation localities, was assignable to *Edmontosaurus* [19, 30–35], Mori et al. [36] recently proposed that the PCF hadrosaurid represented a new and distinct taxon, *Ugrunaaluk kuukpikensis*. However, the taxonomic status of the PCF hadrosaurid remains ambiguous. Xing et al. [37] and Wosik et al. [38] both proposed that *Ugrunaaluk kuukpikensis* is not a valid taxon, because it is based upon disarticulated and disassociated parts from multiple, immature individuals that are not ontogenetically comparable to known specimens of *Edmontosaurus annectens* and *Edmontosaurus regalis*. Furthermore, the recent discovery of lambeosaurine material from the Liscomb Bonebed demonstrated that the PCF bears at least two different kinds of hadrosaurids [10], raising the possibility that *Ugrunaaluk kuukpikensis* could be chimeric. Therefore, reexamination of the PCF hadrosaurine material is mandatory in order to either uphold or refute the taxonomic validity of *Ugrunaaluk kuukpikensis*.

Here we present the first full description of the cranial elements of the PCF hadrosaurine. We focus on cranial material due to the difficulties in distinguishing immature hadrosaurine postcranial elements from those of lambeosaurines [39–42], emphasized especially by the incompleteness of the available postcranial elements. Comparisons of the PCF hadrosaurine cranial material to other hadrosaurines, especially with *Edmontosaurus* may resolve the taxonomic uncertainty of the PCF hadrosaurine. Reexamination of the PCF hadrosaurine from the Arctic also offers the opportunity to understand how latitudinal environmental differences might have influenced hadrosaurid evolution.

### Institutional abbreviations

AMNH FARB, American Museum of Natural History, New York, USA; CMN, Canadian Museum of Nature, Ottawa, Canada; DMNH, Perot Museum of Nature and Science, Dallas, USA; DMNH EPV, Denver Museum of Nature and Science, Denver, USA; NMMNH P, New Mexico Museum of Natural History and Science, Albuquerque, USA; ROM, Royal Ontario Museum, Toronto, Canada; TMP, Royal Tyrrell Museum of Paleontology, Drumheller, Canada; UAMES, University of Alaska Museum, Fairbanks, USA; USNM, United States National Museum, Smithsonian Institution, Washington, DC, USA.

## Materials and methods

As travel funds were a limiting factor for RT during this study, for the purposes of fiscal efficiency, high-quality casts of UAMES specimens that are housed at CMN were included in this study.

### Phylogenetic analyses

Phylogenetic analyses were conducted based on the most recent hadrosauroid data matrix by Kobayashi et al. [43]. In addition to the PCF hadrosaurine, two OTUs (Operational Taxonomic Unit) of immature *Edmontosaurus annectens* (AMNH FARB 5046, an articulated partial skull; and disarticulated juvenile specimens at the Royal Ontario Museum: list of specimens in S2 Table) were added to the data matrix. Four new characters were added to the character list of Kobayashi et al. [43] (S1 Text). The updated data matrix comprises 73 OTUs and 354 equally-weighed, unordered characters (S1 Data). Two phylogenetic analyses, one excluding and one including the two immature *Edmontosaurus annectens* OTUs, were conducted in order to test how maturity of the representative specimens could potentially affect the phylogenetic position of currently recognized *Edmontosaurus* species and the PCF hadrosaurine.

Following previous works [36, 38, 42, 44–46], hadrosauroid ontogenetic variations of character status were evaluated prior to conducting the phylogenetic analyses. Ontogenetically-variable characters were scored as multi-states in the PCF hadrosaurine and the two juvenile *Edmontosaurus* OTUs following Takasaki et al. [42]. Phylogenetic analyses were conducted using TNT ver. 1.5 [47], setting *Ouranosaurus nigeriensis* as the outgroup. The maximum number of trees was set to 99,999 in memory. A traditional search with 1000 replicates of Wagner trees using random additional sequences followed by the TBR branch swapping that held 1000 trees per replicate was performed. Bootstrap resampling using standard absolute frequencies with 5000 replicates and calculation of Bremer decay indices were performed to indicate the support for clades recovered in the resultant trees.

### Disparity analysis

In order to assess the phylogenetic morphological disparity among hadrosaurines, principal coordinate analyses were conducted using the MorphMatrix2PCoA function of the R package Claddis 0.3.0 [48] on R version 3.5.2 [49]. Only the cranial characters were used in the analysis since postcranial elements of the PCF hadrosaurine were not considered in the present phylogenetic analyses. The Maximum Observable Rescaled Distance was utilized for the distance metric. Partial disparity [50] of the phylogenetic morphological disparity were calculated and compared among hadrosaurine subgroups to explore the relative morphological disparity of *Edmontosaurus*. Partial disparity of each subgroup was obtained by dividing the summed square distances of PC 1 through PC 10 of each individual by n-1.

## Results

### Description

When descriptions of cranial elements are based on multiple specimens, the described features are shared among all of the observed specimens unless otherwise mentioned.

### Premaxilla

**Fig 1:**
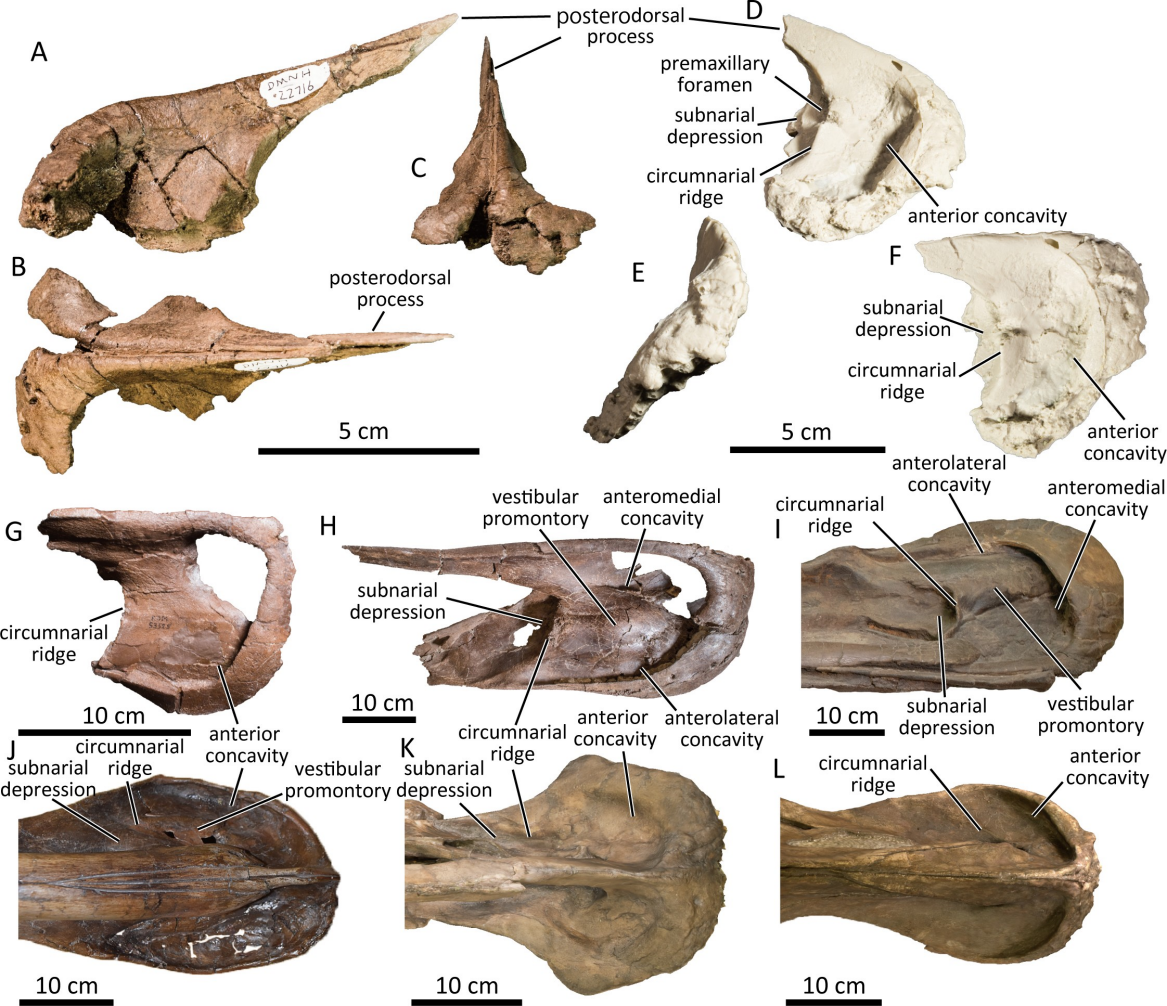
Premaxillae of the PCF hadrosaurine (A-F). DMNH 22716, partial left and right premaxillae in left lateral (A), dorsal (B), and anterior (C) views. UAMES 12995, cast of a partial right premaxilla in right lateral (D), anterior (E), and dorsal (F) views. Premaxillae of immature *Edmontosaurus annectens* ROM 53525(G), mature *Edmontosaurus annectens* ROM 64076 (H), *Edmontosaurus regalis* FMNH P15004 (I), *Prosaurolophus maximus* CMN 2870 (J), *Brachylophosaurus canadensis* CMN 8893 (K), and *Gryposaurus notabilis* CMN 2278 (L) in dorsal views.

The posterodorsal process and the anteromedial margin of the oral rim are preserved in DMNH 22716 (Fig 1A–1C) and the prenarial oral platform is preserved in UAMES 12995 (Fig 1D–1F). The premaxilla bears the bony external naris (DMNH 22716, UAMES 12995), a gracile posterodorsal process (DMNH 22716), and distinct circumnarial ridge (UAMES 12995) as is typical in hadrosaurines (Fig 1). The oral margin of the premaxilla is deflected posterodorsally and forms a prominent lip-like structure (UAMES 12995) that resembles *Edmontosaurus annectens* (e.g., CMN 8509, ROM 57100), *Edmontosaurus regalis* (e.g., CMN 2288, ROM 00801), *Laiyangosaurus youngi* [51], and *Shantungosaurus* [35, 52]. The anterodorsal surface of the lip-like structure is perforated by several small foramina (DMNH 22716, UAMES 12995). The prenarial region anterior to the circumnarial ridge is short relative to the premaxilla width (UAMES 12995) as in *Brachylophosaurus canadensis* (e.g., CMN 8893) *Edmontosaurus regalis* (e.g., CMN 2288), *Gryposaurus notabilis* (e.g., CMN 2278), and immature *Edmontosaurus annectens* (e.g., ROM 53525). The short prenarial region may either represent the immature state in Edmontosaurini [30, 36, 45] or be an indication of an affinity to *Edmontosaurus regalis* [37]. As Mori et al. [36] highlighted, the vestibular promontory is undeveloped in the PCF hadrosaurine premaxilla, thus the circumnarial depression is not divided into anteromedial and anterolateral concavities (UAMES 12995; Fig 1F), which is similar to the condition in a larger but still relatively immature *Edmontosaurus annectens* (ROM 53525; Fig 1G). A premaxillary foramen is present at the medioventral margin of the circumnarial ridge (DMNH 22716, UAMES 12995). The palatal plane is widely angled from the contact surface for its counterpart (UAMES 12995; 135 degrees). The ventral surface of the anterior oral margin of the premaxilla is ornamented by a double-layered denticulate structure (DMNH 22716, UAMES 12995).

### Maxilla

**Fig 2:**
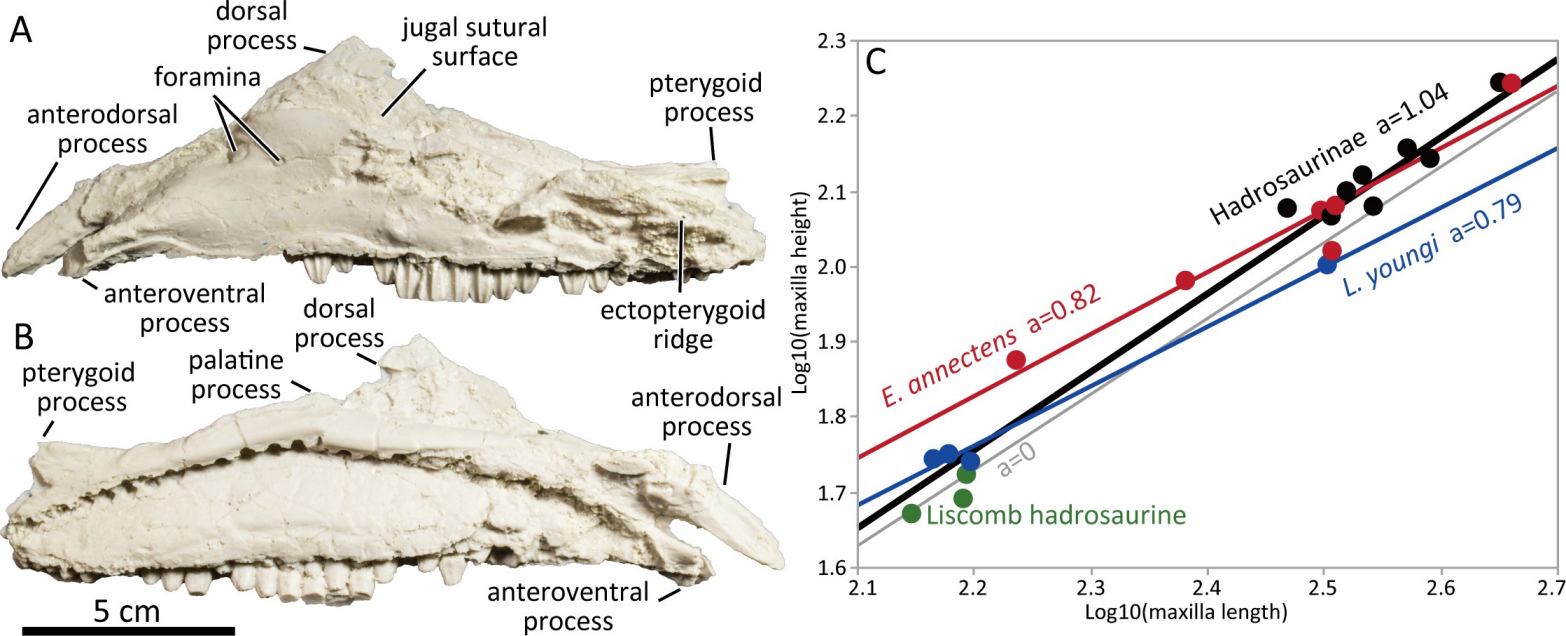
Cast of left maxilla (UAMES 4250) in lateral (A) and medial (B) views. (C) Height-length relationships of hadrosaurine maxillae, demonstrating negative allometry among Edmontosaurini.

The maxilla bears an anterodorsal process and lacks a wide premaxillary shelf as in typical hadrosaurines (Fig 2A and 2B). This differs from lambeosaurine maxillae which lack an anterodorsal process and have a slender dorsal process [53]. The anterodorsal process extends further anteriorly than the anteroventral process. The anteroventral process forms an angle of ~32 degrees with respect to the oral margin of the maxilla in lateral aspect, with some degree of variation (DMNH 22718, DMNH 2014-12-798, UAMES 4250, UAMES 4327). The anteroventral process is straight, unlike those of *Brachylophosaurus canadensis* [54] and *Acristavus gagslarsoni* [55] which are ventrally deflected. Multiple small foramina penetrate the lateral surface of the maxilla ventral to the jugal sutural surface. The lateral exposure of the anterior region of the maxilla anteroventral to the jugal sutural surface is subarcuate. Its dorsal apex is positioned over the anterior one-third of the distance from the anterior end of the maxilla, relative to the oral margin length (e.g., UAMES 4250). The dorsal process is wider than tall and its apex is slightly pointed, unlike the rounded apex of larger *Edmontosaurus annectens* (e.g., ROM 64083) and *Edmontosaurus regalis* (e.g., CMN 2289) specimens, but resembles those of *Acristavus gagslarsoni* [55], *Brachylophosaurus canadensis* [54], and *Naashoibitosaurus ostromi* (NMMNH P-16106). The apex of the dorsal process is located slightly anterior to the midline of the maxilla as in other immature hadrosauroids [45, 56, 57]. Posterior to the sutural surface with the jugal, the ectopterygoid ridge is slightly less than half as long as the maxilla length along the oral margin. The palatine process of the maxilla is dorsally oriented at the posteromedial base of the dorsal flange. The medial surface of the maxilla is perforated by a gently arched row of neural foramina above the mid-height of the main body of the maxilla. The available specimens (DMNH 22718, UAMES 4250, UAMES 4327) suggest there are approximately two alveoli per centimeter, which is expected to decrease with increased ontogenetic development [42, 57]. As Mori et al. [36] pointed out, the maxillae are relatively low, with a height approximately 34% of anteroposterior length measured along the oral margin. This proportion, which is negatively allometric among Edmontosaurini (Fig 2C and S2 Table), resembles that of *Laiyangosaurus youngi* [51].

### Nasal

**Fig 3:**
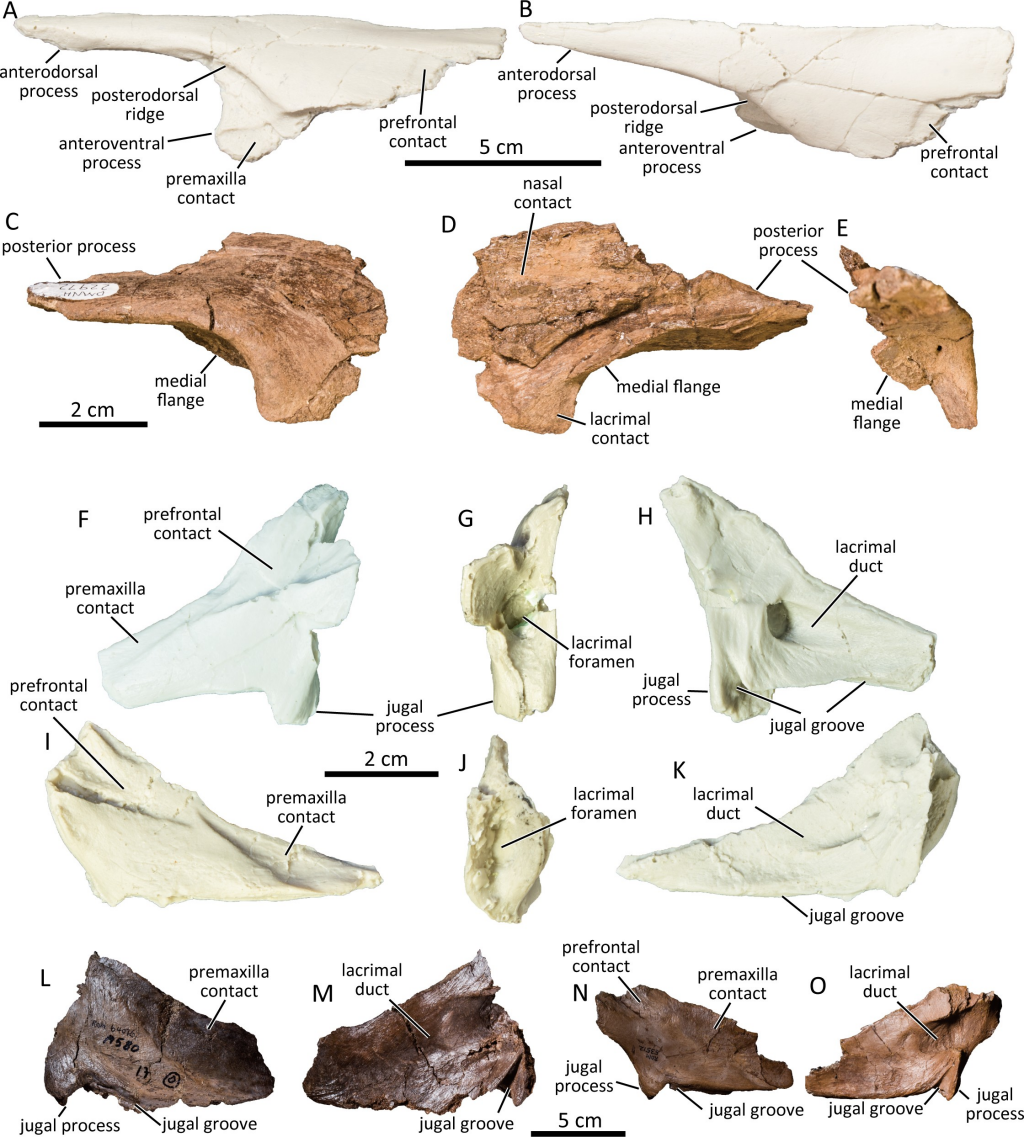
Cast of left nasal (UAMES 4940) in lateral (A) and medial (B) views. Right prefrontal (DMNH 22972) in lateral (C), medial (D), and posterior (E) views. Casts of left (UAMES 4245; F-H) and right (UAMES 4254; I-K) lacrimals the PCF hadrosaurine in lateral (F, I), posterior (G, J), and medial (H, K) views. Isolated right lacrimals of a large (ROM 64076; L, M) and a small (ROM 53512; N, O) *Edmontosaurus annectens* in lateral (L, N) and medial (M, O) views.

The nasal (UAMES 4271) is composed of the elongated anterodorsal process, the main body, and a triangular anteroventral process (Fig 3A and 3B) as in other non-crested hadrosaurines [53, 58]. The anterodorsal process is nearly straight for its entire length. Its cross-section is subtriangular proximally and mediolaterally compressed distally. The dorsoventral height of the anterodorsal process is nearly constant throughout its length and has a blunt distal end (UAMES 4271) unlike the pointed distal end seen in *Gryposaurus notabilis* (CMN 2278, ROM 00873, TMP 1990.022.0001). The medial surface of the anterodorsal process is flat or slightly concave dorsoventrally for contact with its counterpart proximally and with the premaxilla distally. The lateral surface of the anterodorsal process bears a distinct posterodorsal ridge that defines the posterodorsal margin of the circumnarial fossa. The posterodorsal ridge is well-developed as in *Edmontosaurus annectens* (ROM 53523, ROM 53524, ROM 64623), *Edmontosaurus regalis* (CMN 2288, CMN 2289), *Kerberosaurus manakini* [59, 60] and *Shantungosaurus giganteus* [35]. The posterodorsal ridge in the PCF specimens forms a shallow dorsally concave arch which resembles *Kerberosaurus manakini* [59, 60] but differs from large *Edmontosaurus annectens* (e.g., USNM 3814) and *Edmontosaurus regalis* [37] in which the arch is much deeper. This could be an ontogenetically variable feature as demonstrated in the ontogenetic series of *Edmontosaurus annectens* (DMNH EPV. 95227, DMNH EPV. 95234, ROM 53523, ROM 53524, ROM 64076, ROM 64623, USNM 3814).

The triangular anteroventral process projects anteroventrally from the main body of the nasal and is partly covered by the premaxilla laterally. The contact surface with the premaxilla is defined dorsally by the ventral ridge of the main body. The main body of the nasal is trapezoidal and its flat dorsal surface bears a shallow depression posterolaterally for contact with the prefrontal.

### Prefrontal

The prefrontal lacks a posterodorsal process (DMNH 22972; Fig 3C–3E), as in other non-crested hadrosaurines [53]. The anterior preorbital region is mediolaterally thin and anteroposteriorly elongated as in *Edmontosaurus annectens* and *Edmontosaurus regalis* [37]. The anterior margin of the prefrontal is fan-shaped, unlike the nearly square anterior margins of large *Edmontosaurus annectens* (e.g., ROM 64076) and *Edmontosaurus regalis* (e.g., CMN 2289). Since the anterior margins of the prefrontal of relatively immature *Edmontosaurus annectens* are bifurcated (ROM 53499, ROM 53500), the feature in the PCF hadrosaurine may vary ontogenetically, although it is currently untestable. The lateral surface of the preorbital region is smooth in DMNH 22972 but is penetrated by several foramina in some other specimens (e.g., UAMES 4305). The orbital margin forms a smooth right-angle anterodorsally, unlike the gently arcuate anterodorsal orbital margins of *Brachylophosaurus canadensis* (CMN 8893), *Maiasaura peeblesorum* [61], and *Saurolophus osborni* (AMNH FARB 5221, CMN 8796), but similar to *Edmontosaurus annectens* and *Edmontosaurus regalis* [35, 37] including immature specimens (e.g., ROM 53499, ROM 53500). The anteroventral surface of the orbital margin is flat and the medial flange is undeveloped as in hadrosaurines including immature *Edmontosaurus annectens* specimens (e.g., ROM 53499, ROM 53500) and *Kerberosaurus manakini* [59, 60]. There is no deep circular fossa (associated with postorbital pocket [30, 35]) along the lateral half of the posteroventral surface of the prefrontal, which is a diagnostic feature of *Edmontosaurus* [35]. The prefrontal tapers posteriorly and forms a wedge-shaped posterior process.

### Lacrimal

The lacrimals (UAMES 4245, UAMES 4254) are much longer anteroposteriorly than its dorsoventral height (Fig 3F–3K), unlike lambeosaurine lacrimals which are generally dorsoventrally higher than anteroposteriorly long. It is anteroposteriorly elongated, subtriangular, and tapers anteriorly to a point (UAMES 4254) as in the lacrimals of *Maiasaura peeblesorum* [61] and *Brachylophosaurus canadensis* [62, 63]. Unlike the lacrimals of large specimens of *Edmontosaurus annectens* (ROM 53511, ROM 53512, ROM 64076) and *Edmontosaurus regalis* [37], contact surfaces with the premaxilla and prefrontal are dorsoventrally short (UAMES 4245, UAMES 4254). The lacrimal foramen opens into the orbital rim and the lacrimal duct is enclosed medially by a thin plate of bone (UAMES 4245) as in *Edmontosaurus regalis* [37] and *Probrachylophosaurus* [62], but unlike in *Edmontosaurus annectens* in which the duct is open medially (Fig 3L–3O). The ventral margin of the lacrimal has a deep longitudinal groove for contact with the anterior process of the jugal (UAMES 4245). The jugal process protrudes ventrally from the posterolateral margin of the lacrimal and is nearly at right angles to the jugal groove (UAMES 4245).

### Jugal

**Fig 4:**
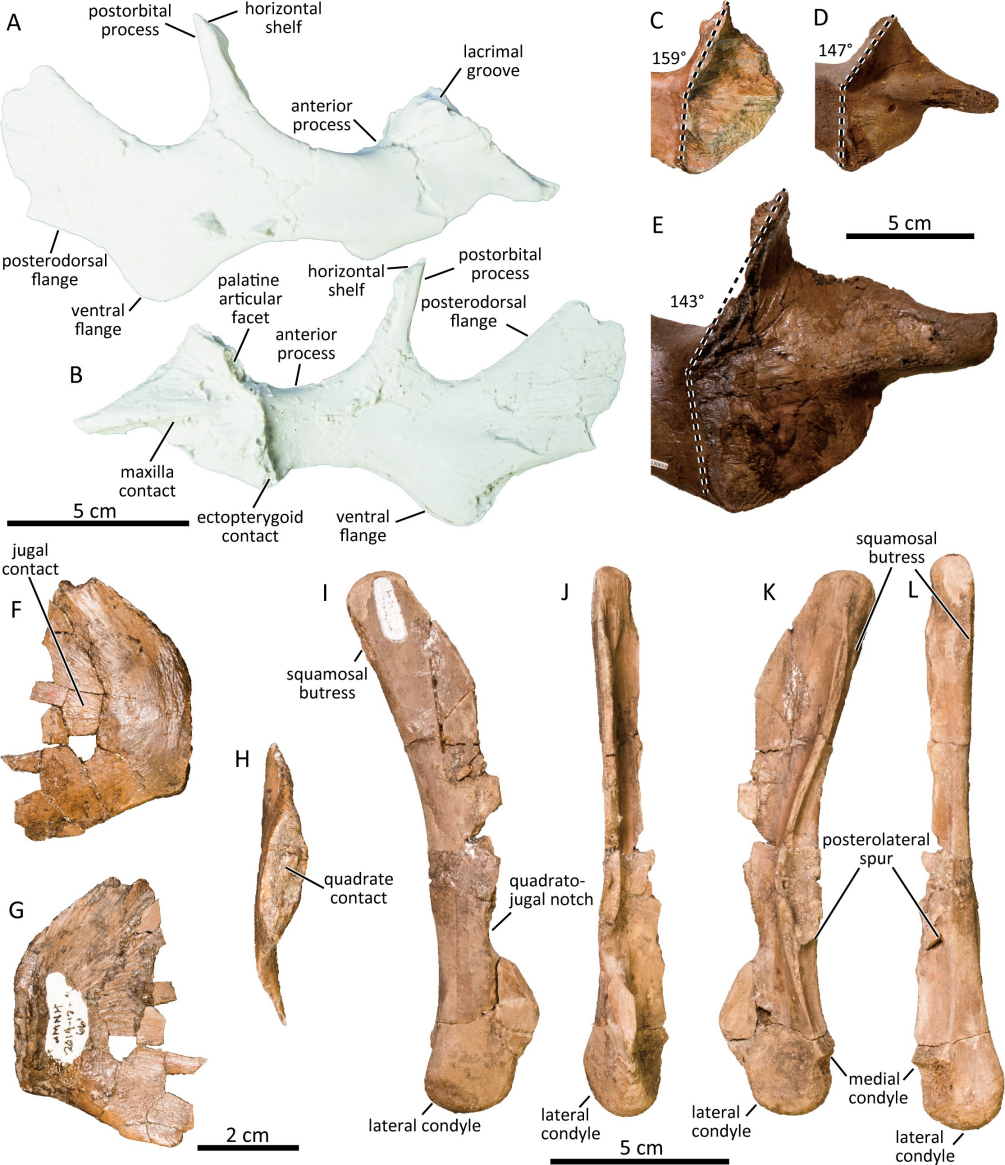
Cast of right jugal of the PCF hadrosaurine (UAMES 4187) in lateral (A) and medial (B) views. Anterior process of left jugal of *Edmontosaurus annectens* (C: ROM 53518; D: DMNH EPV. 95220; E: DMNH EPV. 130653) in medial view, demonstrating intraspecific variation of the angle between the palatine process and the posterior margin of the maxilla contact surface. Left quadratojugal (DMNH 2014-12-661) in lateral (F), medial (G), and posterior (H) views. Right quadrate (DMNH 21044) in lateral (I), anterior (J), medial (K), and posterior (L) views.

A right jugal UAMES 4187 is nearly complete (Fig 4A and 4B). Its anterior process is dorsoventrally expanded as in other hadrosaurids. The anterior end of the anterior process forms a long, wedge-shaped anterior ‘spur’ as in typical hadrosaurines [53] slightly dorsal to the mid-height of the anterior process. The palatine articular facet is anterodorsally inclined at an angle between 125° – 136° from the line connecting the ventral apices of the infratemporal fenestra and the orbit. Mori et al. [36] proposed that the angle between the palatine articular facet and the posterior margin of the maxilla contact surface of the jugal of the PCF hadrosaurine is greater than that of *Edmontosaurus annectens* of comparable size, and they proposed that a hypothetical ontogenetic trajectory of this feature would be distinct from that of *Edmontosaurus*. However, additional observations of *Edmontosaurus annectens* jugals demonstrate a high degree of intraspecific variation in this angle regardless of size (Fig 4C–4E). Furthermore, the jugal of *Kerberosaurus manakini*, another member of Edmontosaurini, also has an anterodorsally inclined palatine process [59, 60]. Therefore, this feature is not unique to the PCF hadrosaurine. The dorsal contact surface with the lacrimal bears a longitudinal groove. The contact surface widens mediolaterally along the posterodorsal margin, but does not form the posterolateral projection along the orbital margin that is present in *Edmontosaurus regalis* [37]. The ventral margin of the anterior process is pointed as in several immature *Edmontosaurus annectens* specimens (e.g., DMNH EPV. 95220).

The postorbital process of the jugal is inclined ~73 degrees posterodorsally relative to the line connecting the ventral apices of the infratemporal fenestra and the orbit. The postorbital process bears the horizontal shelf on the postorbital process of the jugal as in *Edmontosaurus regalis* [37]. The ventral flange of the jugal is well-developed and ventrally rounded as in *Edmontosaurus annectens* (e.g., ROM 57100), *Edmontosaurus regalis* (e.g., AMNH FARB 5254), *Kamuysaurus japonicus* [43], and *Kerberosaurus manakini* [59, 60]. The ventral flange is approximately 1.24 times the height of the posterior constriction ventral to the infratemporal fenestra, and the posterior constriction is approximately 0.64 times the height of the length between the ventral apexes of the orbit and the infratemporal fenestra (UAMES 4187, UAMES 7336). The posteroventral margin of the posterodorsal flange of the jugal is slightly concave and divergent from the anterodorsal margin of the posterodorsal flange.

### Quadratojugal

The quadratojugal is flat and slightly bowed laterally (Fig 4F–4H). Its posteroventral corner is pointed (DMNH 2014-12-661, DMNH 2014-12-183, UAMES 4272, UAMES 21537) as in *Edmontosaurus regalis* (e.g., AMNH FARB 5254), but is different from the strongly hooked posteroventral corners of *Brachylophosaurus canadensis* (e.g., CMN 8893) and *Maiasaura peeblesorum* (e.g., ROM 44770). The anterior half of the lateral surface is slightly depressed, forming the jugal contact. The quadrate contact faces posteriorly in some (e.g., DMNH 2014-12-661) and posteromedially in other specimens (e.g., DMNH 2014-12-813), demonstrating some morphological variation within the sample, which may represent the unintended inclusion of lambeosaurine quadratojugals in the studied sample.

### Quadrate

The quadrate (DMNH 2014-12-744, DMNH 21044, UAMES 2486, UAMES 4235) is bowed (concave posteriorly; Fig 4I–4L) as in immature *Edmontosaurus annectens* (e.g., ROM 53522), *Saurolophus angustirostris* [64], and lambeosaurines [53]. The squamosal buttress is faint, unlike the well-developed squamosal buttress of *Gryposaurus notabilis* (TMP 1980.022.0001) and *Brachylophosaurus canadensis* (CMN 8893). A symmetrical, arcuate quadratojugal notch is located well below mid-height of the quadrate, unlike in lambeosaurines. The quadratojugal notch is shallow and barely reaches half of the anteroposterior depth of the lateral flange. The quadratojugal notch is dorsoventrally asymmetrical, being deeper ventrally than dorsally. The posterodorsal margin of the quadratojugal notch is shallowly grooved. The lateral surface of the quadrate dorsal to the quadratojugal notch lacks a large depression for the lateral scarf joint connection with the quadratojugal, as seen in nestling *Edmontosaurus annectens* [38] but unlike larger specimens of *Edmontosaurus annectens* (e.g., ROM 53522, ROM 64076). The posterolateral spur is located slightly dorsal to the posterior apex of the quadratojugal notch. A groove for contact with the posteroventral projection of the quadrate ramus of the pterygoid is present anterior to the posterolateral spur. Ventrally, the medial condyle is reduced and positioned dorsal to the lateral condyle as in other hadrosaurids. The lateral condyle is slightly longer anteroposteriorly than mediolateral width of the ventral end of the quadrate in some specimens (e.g. DMNH 2014-12-567), resembling to nestling *Edmontosaurus annectens* [38], and is significantly shorter in some other specimens (e.g., UAMES 4235).

### Squamosal

**Fig 5:**
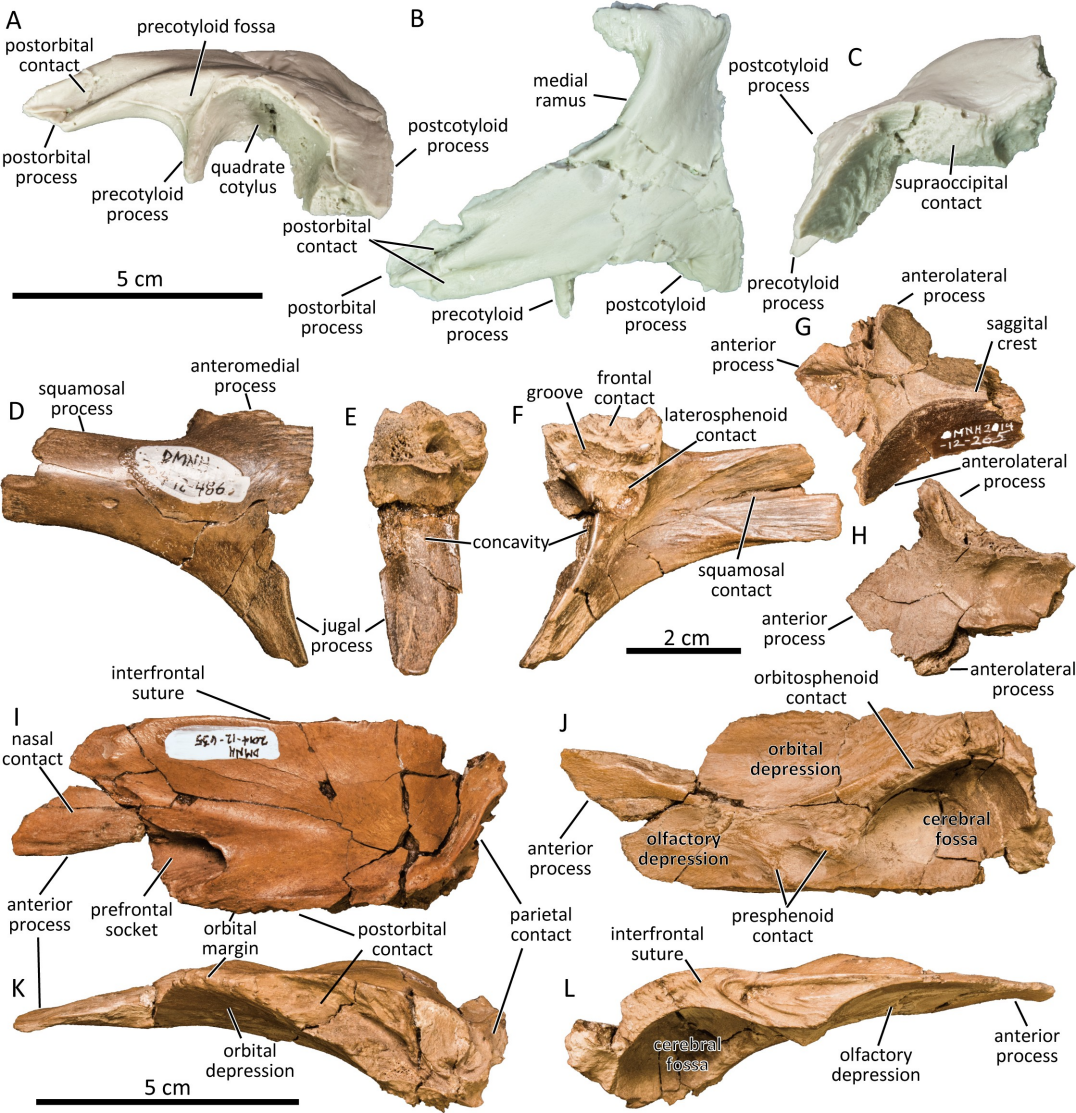
Cast of left squamosal (UAMES 12266) in lateral (A), dorsal (B), and posterior (C) views. Right postorbital (DMNH 2014-12-486) in lateral (D), anterior (E), and medial (F) views. Parietal (DMNH 2014-12-265) in dorsal (G) and ventral (H) views. Left frontal (DMNH 2014-12-635) in dorsal (I), ventral (J), lateral (K), and medial (L) views.

The squamosal is dorsoventrally short (UAMES 12266; Fig 5A–5C) as in typical hadrosaurines [65, 66]. The two deep grooves for contact with the squamosal process of the postorbital reach posteriorly to the level of the precotyloid process. The precotyloid process is triangular in cross section and is as long as the anteroposterior length of the quadrate cotylus, as also seen in immature specimens of *Edmontosaurus annectens* (e.g., ROM 53510). The precotyloid fossa is triangular in lateral aspect and terminates anterior to the dorsal apex of the quadrate cotylus. The postcotyloid process is massive and its posteroventral surface is striated for contact with the exoccipital paroccipital process. The medial ramus is straight and defines the posterior margin of the supratemporal fenestra. Its medial surface is extremely rugose for contact with the parietal.

### Postorbital

The postorbital is a triradiate bone composed of anteromedial, squamosal, and jugal processes (Fig 5D–5F). The dorsal surface of the postorbital is flat and lacks the dorsal promontorium and the deep depression above the jugal process is present in derived lambeosaurines [67]. The orbital margin of the postorbital is deeply arcuate. The anteromedial process of the postorbital is short, with a medial surface for contact with the frontal that is rugose and bears an anteroposterior groove that separates the triangular laterosphenoid facet with the frontal contact. The squamosal process bifurcates posteriorly (UAMES 4983). The articular surface for the squamosal reaches the proximal base of the squamosal process. The jugal process is slender unlike in *Shantungosaurus giganteus* [35] and is shallowly concave anteriorly. The anterior surface of the jugal process does not form a deep pocket, unlike in large specimens of *Edmontosaurus annectens* (e.g., CMN 2288) and especially of *Edmontosaurus regalis* (e.g., USNM 3814). Although Mori et al. [36] emphasized that the absence of the deep pocket differentiates the PCF hadrosaurine from *Edmontosaurus*, this feature changes drastically through ontogeny in *Edmontosaurus* [30, 37]. The anterior concavity of the PCF hadrosaurine (UAMES 33308) is certainly much shallower compared to the deep pocket in an equivalent sized postorbital of *Edmontosaurus annectens* (ROM 53513) [36]. However, the anterior concavity of UAMES 33308 is also relatively much wider than the pocket of the immature *Edmontosaurus annectens*. Since postorbital pocket of *Edmontosaurus regalis* is much wider than that of *Edmontosaurus annectens* [37], the wide anterior concavity of the PCF hadrosaurine may resemble an immature *Edmontosaurus regalis*, although the absence of an equivalently-immature *Edmontosaurus regalis* postorbital hinders further comparisons. As a result, it is impossible to confirm either the presence or absence of this character in *Edmontosaurus regalis*, and hence renders its utility as a diagnostic character of the PCF taxon highly suspect.

### Parietal

The anterior half of the parietal is known (Fig 5G and 5H). The dorsal exposure of the anterior process of the parietal is mediolaterally narrow and finger-shaped as in *Edmontosaurus* and *Shantungosaurus giganteus* [35, 37]. The dorsal surface of the parietal posterior to the anterior process is flat and is bounded by arched ridges that extend posteriorly from the anterolateral processes. The ridges merge posteromedially and are continuous with the straight sagittal crest. Ventrally, the parietal is mediolaterally concave and forms the posterior part of the cerebral fossa.

### Frontal

The frontal of the PCF hadrosaurine is nearly twice as long as it is mediolaterally wide (DMNH 2014-12-635, Fig 5I–5L), unlike the short frontals of lambeosaurines and crested hadrosaurines [53]. The dorsal surface of the frontal is flat medially and slightly elevated laterally as in *Edmontosaurus annectens*, *Edmontosaurus regalis*, and *Shantungosaurus giganteus* [35, 37]. Dorsally, the contact surface for the nasal is anteromedial to the socket for posterior process of the prefrontal, as in members of Edmontosaurini (except *Kamuysaurus japonicus* [43]). The entire posterior margin of the frontal is rugose for contact with the parietals. Posterolaterally, the postorbital suture is oriented nearly parallel to the interfrontal suture. Anterior to the postorbital suture, the frontal participates in the dorsal margin of the orbit as other members of Edmontosaurini [35] including *Kerberosaurus manakini* and *Shantungosaurus giganteus* [35, 59, 60]. The frontal orbital margin is more than 20% as long as the interfrontal suture length. The dorsal plate of the frontal is thin anteriorly above the olfactory depression and thick posteriorly above the cerebral fossa. The olfactory depression is shallow and anteroposteriorly elongated and is slightly concave mediolaterally. It is separated from the orbital depression by a low ridge. The cerebral fossa is the deepest among the three depressions and is semi-ovoid that is wider and deeper posteriorly than anteriorly. The presphenoid contact is located in between the olfactory depression and the cerebral fossa.

### Pterygoid

**Fig 6:**
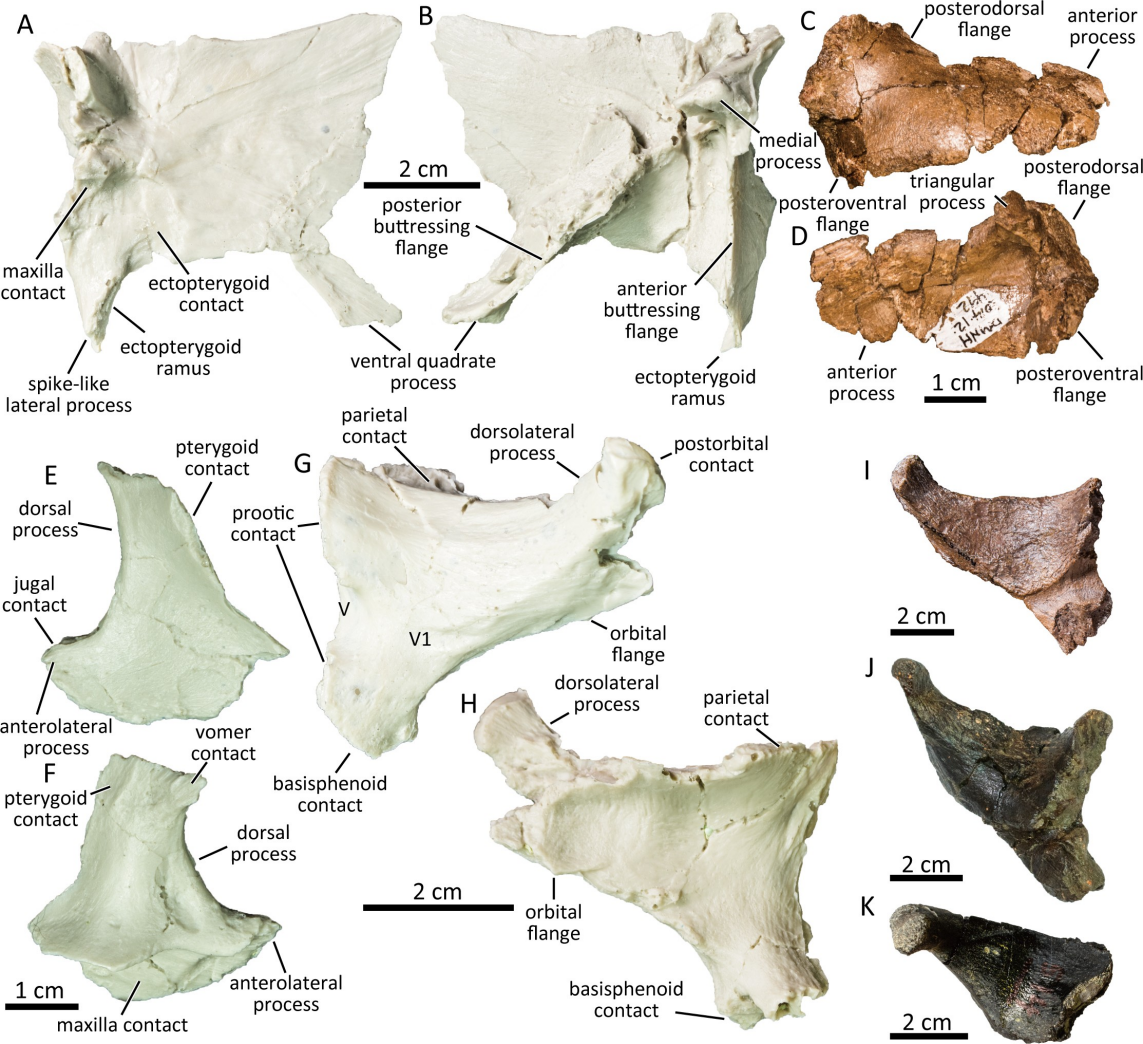
Cast of left pterygoid (UAMES 4215) in lateral (A) and medial (B) views. Right ectopterygoid (DMNH 2014-12-492) in lateral (C) and medial (D) views. Cast of left palatine (UAMES 4257) in lateral (E) and medial (F) views. Cast of right laterosphenoid of the PCF hadrosaurine (UAMES 4352) in lateral (G) and medial (H) views. Laterosphenoids of *Edmontosaurus annectens* (I; ROM 53507), *Hypacrosaurus stebingeri* (J; USNM 11893), and *Eolambia caroljonesa* (K; CEUM 34366) in lateral view.

The palatine process and the distal half of the dorsal quadrate process are missing in the available pterygoid (UAMES 4215; Fig 6A and 6B). The lateral surface of the main body of the pterygoid is flat posteriorly and shallowly depressed anteriorly for the ectopterygoid contact as in typical hadrosaurines [68]. The maxilla contact anterior to the ectopterygoid contact surface is deeply incised to interlock the pterygoid process of the maxilla. Ventrally, the ectopterygoid ramus is anteroposteriorly compressed and bears a spike-like lateral process. The ventral ridge in between the ectopterygoid ramus and the ventral quadrate process is straight and well-developed. Medially, the anterior and the posterior buttressing flanges form an angle of 40–45 degrees as in *Edmontosaurus regalis* [68] and meet dorsally at the medial process.

### Ectopterygoid

The anterior process of the ectopterygoid (DMNH 2014-12-492, UAMES 4240) is dorsoventrally thin (Fig 6C and 6D). The posterodorsal flange and the posteroventral flange flare laterally, forming a crescent shaped posteromedial surface that articulates with the pterygoid. The posterodorsal and the posteroventral flanges of the ectopterygoid are small, resembling the condition in relatively immature *Edmontosaurus annectens* (e.g., ROM 53505). The posteroventral process is recurved ventrally to overlap the posterodorsal surface of the maxilla. Medially, the triangular process, a feature present in members of Hadrosaurinae [68], is located at the junction between the anterior process and the posterodorsal flange. The triangular process articulates with the main body of the pterygoid and the pterygoid process of the maxilla.

### Palatine

The dorsal process of the palatine is robust (UAMES 4257; Fig 6E and 6F), unlike those of lambeosaurines (e.g., CMN 8633), hook-shaped and anterodorsally tapering. The dorsal process is shallowly concave laterally and convex medially. The medial surface of the dorsal hook has a striated contact for the vomer anteriorly and the pterygoid contact posteriorly. Ventrally, the contact surface with the maxilla is characterized by a deep anteroposterior groove. The anterolateral process and the jugal contact surface are less developed compared to those of specimens attributable to mature *Edmontosaurus regalis* [37].

### Laterosphenoid

The laterosphenoid (UAMES 4352, UAMES15284) is a subtriangular bone composed of the dorsolateral process, the orbital flange, and the temporal plate (Fig 6G and 6H). The dorsolateral process is short and stout, being less than 70% the length of the temporal plate (68.9% in UAMES 4352 and 57.1% in UAMES 15284) as in *Edmontosaurus* (e.g., ROM 53507; Fig 6I). In all other hadrosauroids besides *Edmontosaurus* this ratio is larger than 90% [37] (Fig 6J and 6K), including a hadrosaurine *Prosaurolophus* [65]. The short dorsolateral process of the laterosphenoids of the PCF hadrosaurines demonstrates the strong affinity of these specimens to *Edmontosaurus*. The distal end of the dorsolateral process is ovoid in articular view, and bears a smooth surface for the articulation with the laterosphenoid fossa of the postorbital. The orbital flange is directed medially to contact with the orbitosphenoid. Laterally, a weak anteroventral ridge separates the orbital flange from the dorsolateral process and the temporal plate. The temporal plate is dorsoventrally concave medially and forms the lateral braincase wall. The posterior margin of the laterosphenoid bears the anterior half of the foramen for the trigeminal nerve (CN V). The wide horizontal groove for the ophthalmic branch (CN V1) extends anteriorly from the foramen for the trigeminal nerve. The region between the anterior margin of the trigeminal nerve and the anteroventral ridge is short, and associated with the anteroposteriorly short contact with the basisphenoid. The region is anteroposteriorly as long or shorter than the dorsoventral height of the trigeminal nerve as in *Edmontosaurus annectens* (CMN 8509, ROM 53507, ROM 53508, AMNH FARB 427, AMNH FARB 59786, AMNH FARB 64623), *Edmontosaurus regalis* (CMN 2288), and *Saurolophus osborni* (AMNH FARB 5221), but unlike in *Brachylophosaurus canadensis* (CMN 8893), *Gryposaurus notabilis* (AMNH FARB 5350), and *Maiasaura peeblesorum* (ROM 66181) in which the region is much longer. The passage for the mandibular branch (CN V3) is absent in the available laterosphenoids of the PCF hadrosaurines.

### Prootic

**Fig 7:**
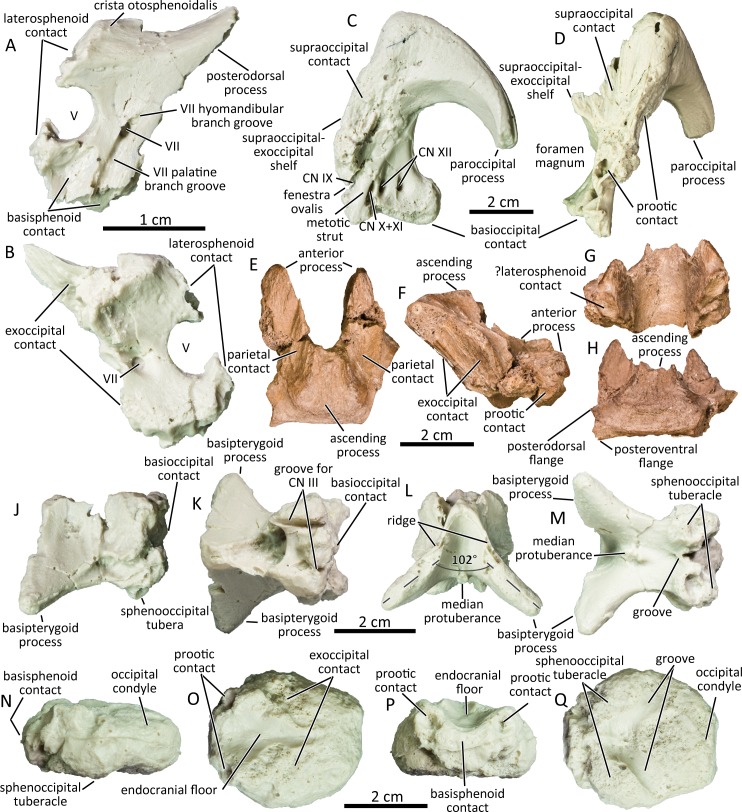
Cast of left prootic (UAMES 4357) in lateral (A) and medial (B) views. Cast of left exoccipital (UAMES 4263) in lateral (C) and anterior (D) views. Supraoccipital (DMNH 22807) in dorsal (E), right lateral (F), anterior (G), and posterodorsal (H) views. Cast of basisphenoid (UAMES 4301) in left lateral (J), dorsal (K), anterior (L), and ventral (M) views. Cast of basioccipital (UAMES 34723) in left lateral (N), dorsal (O), anterior (P), and ventral (Q) views.

The main body of the prootic defines the dorsal, posterior, and ventral margins of the trigeminal foramen (UAMES 4357; Fig 7A and 7B). Posteriorly, the anterodorsal margin of the fenestra ovalis is unclear in the available specimens. The foramen for the facial nerve (CN VII) pierces the prootic posterior to the trigeminal foramen. The foramen for the facial nerve is contiguous with two grooves that run parallel to the posterior margin of the prootic along the lateral surface. The shorter, less defined groove that runs posterodorsally from the foramen for the facial nerve conducted the hyomandibular branch of the facial nerve, and the longer groove that runs anteroventrally from that same foramen accommodated the palatine branch [66]. On the dorsolateral surface of the prootic, the crista otosphenoidalis is more developed than in *Brachylophosaurus canadensis* (CMN 8893) and resembles those of *Edmontosaurus annectens* (CMN 8509) and *Edmontosaurus regalis* (CMN 2289). The lateral surface ventral to the trigeminal foramen is excavated to form a scarf joint with the alar process of the basisphenoid. The mediolaterally thin posterodorsal process of the prootic contacts the exoccipital medially and the parietal dorsally.

### Exoccipital

The supraoccipital-exoccipital shelf is moderately elongated posteriorly unlike in lambeosaurines which are extremely reduced [10]. The anterior margin of the occipital plate defines the posterior half of the common opening for the fenestra ovalis and the adjacent metotic foramen for the glossopharyngeal nerve (CN IX) [69, 70]. Posterodorsal to the fenestra ovalis, the lateral surface of the exoccipital is rugose where it contacts the prootic. The metotic strut is located posterior to the fenestra ovalis. Posterior to the metotic strut, three foramina pierce the occipital plate. The anterior large foramen represents the common opening for the vagus nerve (CN X) and the accessory nerve (CN XI) [37, 69–71]. The posterior two foramina represent the openings for separate branches of the hypoglossal nerve (CN XII). The ventral margin of the exoccipital for contact with the basioccipital is slightly sinuous. The condyloid is subtriangular in occipital view as in immature *Edmontosaurus* [37]. The paroccipital process is directed ventrolaterally and gradually tapers to a blunt tip. The paroccipital process does not curve anteriorly unlike in larger specimens of *Edmontosaurus* [30, 37].

### Supraoccipital

The hadrosaurine supraoccipitals (DMNH 22807, UAMES 4291, UAMES 12727 UAMES 21544) lack squamosal bosses and are anteroposteriorly elongated (Fig 7E–7H), unlike in lambeosaurines including a recently-described lambeosaurine supraoccipital from the Liscomb bonebed locality [10]. The dorsolateral surfaces of the supraoccipital are excavated for contact with the posterolateral processes of the parietals. The excavations are deep in large specimen (UAMES 12727) as in juvenile *Edmontosaurus annectens* (ROM 53494), but shallower in smaller specimens (UAMES 4291, UAMES 21544). The ascending process is wide and low in small specimens and becomes narrower and taller in large specimens, occupying much less than the maximum mediolateral width of the supraoccipital. The posterior surface of the ascending process is markedly inclined anterodorsally. The anteromedial surface of the supraoccipital defines the smooth posterior surface of the endocranial cavity. The ventrolateral surface of the anterior process is triangular and contacts with the posterodorsal process of the prootic [65]. Whether there is contact with the laterosphenoid at the anterior end of the supraoccipital, as in *Prosaurolophus maximus* [65], is unclear in the PCF hadrosaurines. Posteriorly, the lateroventral and the ventral surfaces of the supraoccipital are striated for receipt of the exoccipital. The posterior surface of the supraoccipital is smooth and has a straight ventral margin. The lateral projections for squamosal contact present in *Prosaurolophus* maximus [65] and *Gryposaurus notabilis* [72] are undeveloped.

### Basisphenoid–parasphenoid complex

The basipterygoid processes of the basisphenoid (UAMES 4301; Fig 7J–7M) are ventrolaterally directed, diverging from each other in anterior/posterior views at an angle of slightly greater than 100 degrees. The angle resembles other hadrosaurines including *Brachylophosaurus canadensis* (CMN 8893), *Edmontosaurus annectens* (AMNH FARB 5046, CMN 8509), *Edmontosaurus regalis* [37], and *Prosaurolophus maximus* [65], but is unlike lambeosaurines in which the angle is less than 100 degrees (e.g., *Corythosaurus casuarius*, ROM 776; *Parasaurolophus tubicen*, NMMNH P25100) [73]. Each basipterygoid process is subtriangular in cross-section and terminates in a blunt point. The anterior margin of each basipterygoid process gradually narrows anterodorsally to a ridge. The cultriform process is not preserved in the available specimens. The median protuberance is present in between the basipterygoid processes. The transverse ridge is absent in the PCF basisphenoid UAMES 34723 as in derived lambeosaurines (e.g., *Hypacrosaurus altispinus*), suggesting that UAMES 34723 could possibly belong to a member of Lambeosaurinae. This feature could also be individually variable, as in some *Edmontosaurus annectens* (present in ROM 59786, absent in AMNH FARB 00427). The posterior surface for contact with the basioccipital is extremely rugose. The bulbous posterior region of the basisphenoid forms the anterior parts of the paired sphenooccipital tubera, which are less developed than those of other hadrosaurines such as *Brachylophosaurus canadensis* (CMN 8893) and larger *Edmontosaurus annectens* (CMN 8509). The sphenooccipital tubera are likely to develop ontogenetically as they fuse with the basioccipital. The sphenooccipital tubera are separated ventrally by a shallow groove at the midline. Two parallel grooves run anteroposteriorly along the dorsal surface of the sphenooccipital tubera for the oculomotor nerve (CN III) [65].

### Basioccipital

In dorsal aspect, the basioccipital is a sub-circular bone that is slightly longer anteroposteriorly than mediolaterally wide (UAMES 34723; Fig 7N–7Q). It is of almost constant dorsoventral thickness. Its dorsal surface bears a wide longitudinal groove forming the ventral surface of the endocranial cavity. The dorsal surfaces lateral to the endocranial cavity are rugose for contact with the exoccipital. The deep depressions located at the anterolateral corners of the basioccipitals are likely the prootic contacts as in *Prosaurolophus maximus* [65]. On the ventral surface of the anterior half of the basioccipital, two prominent protuberances represent the posterior region of the sphenooccipital tubera. Two posterolaterally directed grooves are located between the tubera and the occipital condyle. Posteriorly, the occipital condyle is positioned on the same level with the rest of the basioccipital, as in the isolated basioccipital of an immature *Edmontosaurus annectens* (ROM 53538) but is less angled than in larger *Edmontosaurus annectens* (e.g., ROM 64623).

### Predentary

**Fig 8:**
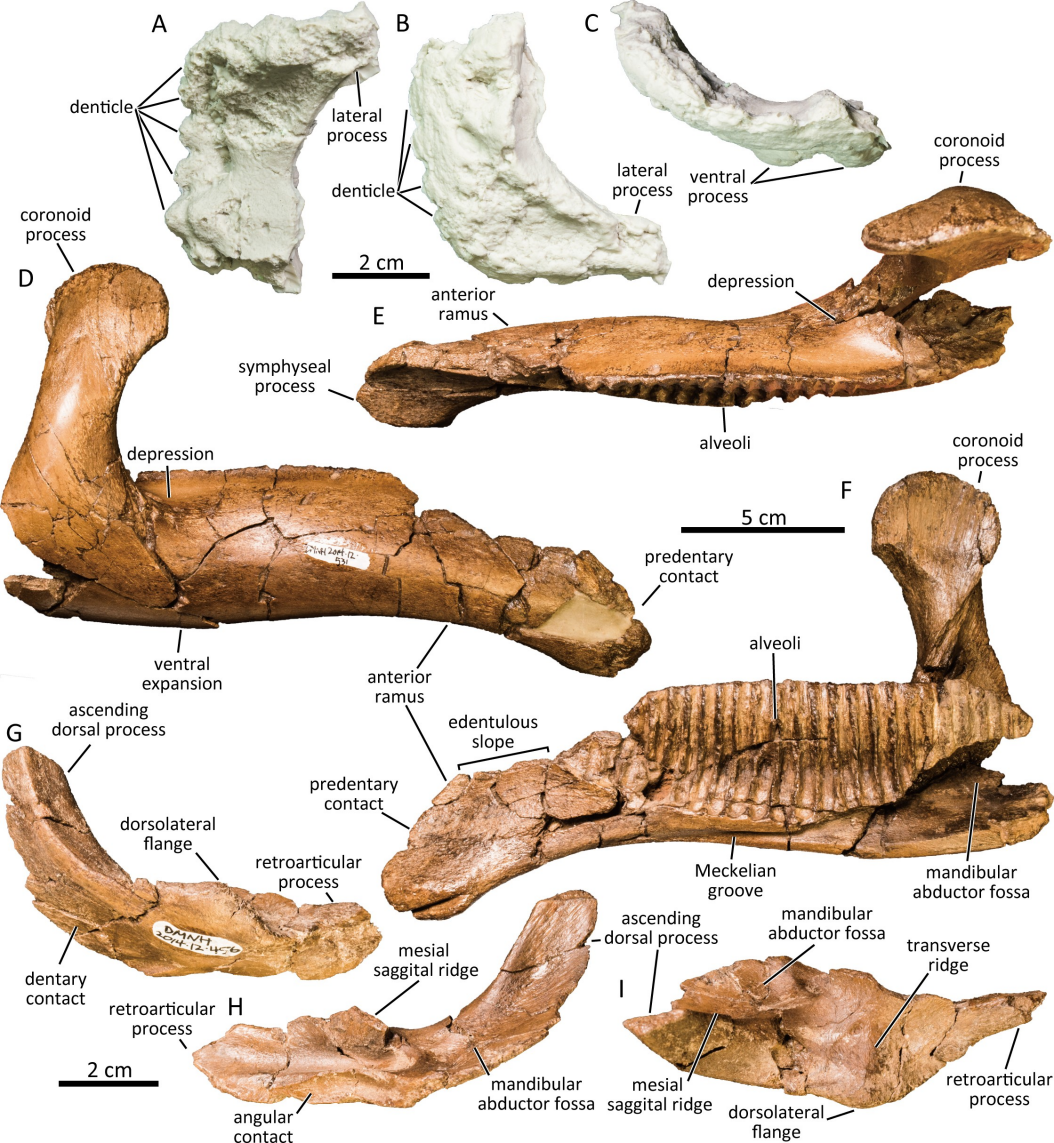
Cast of a partial predentary (UAMES 4437) in dorsal (A), ventral (B), and anterior (C) views. Right dentary (DMNH 2014-12-531) in lateral (D), dorsal (E), and medial (F) views. Left surangular (DMNH 2014-12-456) in lateral (G), medial (H), and dorsal (I) views.

The right half of the anterior ramus of the predentary is preserved (UAMES 4437; Fig 8A–8C). The anterior edge of the predentary is denticulated with four trapezoidal projections and one subtriangular midline projection. Unlike the spoon-shaped predentary of *Kritosaurus* sp.[74], the predentary of the PCF hadrosaurine is flat and shovel shaped with a nearly right-angled anterolateral corner as in *Edmontosaurus regalis* (CMN 2289). The median dorsal process is not preserved. The bi-lobed ventral process at the posteroventral margin of the predentary diverges at its base and is much less developed compared to the predentary of larger *Edmontosaurus annectens* (e.g., USNM 3814).

### Dentary

Multiple nearly complete dentaries are preserved (e.g., DMNH 2014-12-531; Fig 8D–8F). The main body of the dentary is approximately four times as anteroposteriorly long (from anterior end to the posterior margin of the coronoid process) as its dorsoventral height at the midpoint of the dental battery. The bowed expansion ventral to the coronoid process and the ventral deflection of the anterior ramus gives the dentary a slightly sinuous outline in lateral view. The ventral deflection of the anterior ramus forms an angle of about 23 degrees relative to the alveolar margin. The degree of deflection is much stronger than in large *Edmontosaurus annectens* and *Edmontosaurus regalis* (e.g., ROM 46259, CMN 2289), but resembles immature specimens of *Edmontosaurus annectens* (e.g., ROM 53529, ROM 53530). The symphyseal process of the dentary is gently curved lingually and is generally 1.5 times as mediolaterally wide as the minimum mediolateral width of the dentary immediately posterior to it, unlike in lambeosaurines which have wider symphyseal process [73]. The anterodorsal margin of the predentary contact is dorsally convex and descends gradually in lateral view unlike in *Brachylophosaurus canadensis* (CMN 8893) which is strongly concave. The lengths of the proximal edentulous margins vary from 12% to 17% of the length from the anterior end of the alveoli to the posterior margin of the coronoid process in the available specimens. These values are lower than those of larger but still immature individuals of *Edmontosaurus annectens* (e.g., ROM 53529, AMNH FARB 5730) as Mori et al. [36] demonstrated, but are expected to increase during ontogeny [38]. The dentary tooth row bears no less than 1.5 alveoli per centimeter, each of which are separated by sheet-like septa. The alveoli are narrow and aligned perpendicular to the dorsal alveolar margin of the dentary in the posterior half, whereas they are slightly inclined posteriorly in the anterior half of the dental battery. The posterior-most alveolus is positioned posterior to the caudal margin of the coronoid process. The coronoid process is slightly inclined anteriorly in various angles between 75–85 degrees. Its apex is subcircular, lacking the sharp dorsal projection present in *Brachylophosaurus canadensis* (CMN 8893) and some specimens of *Edmontosaurus annectens* (AMNH FARB 5730, ROM 53530). The anteroposterior expansion of the coronoid process is more developed anteriorly than posteriorly as in immature *Edmontosaurus annectens* (e.g., ROM 53529, ROM 53530). The base of the coronoid process is widely separated from the dentary dental battery by a deep depression unlike in a nestling *Edmontosaurus annectens* [38]. The Meckelian groove runs anteroposteriorly along the ventral margin of the dentary, fading out at the ventral deflection of the anterior ramus.

### Surangular

The surangular is composed of the mediolaterally-thin ascending dorsal process, the dorsoventrally-compressed dorsolateral flange, and the mediolaterally compressed retroarticular process (Fig 8G–8I). The ventrolateral surface of the ascending process is shallowly excavated for contact with the dentary. The contact surface reaches to the level of the lateral apex of the dorsolateral flange. The ascending dorsal process is excavated dorsomedially to form the mandibular abductor fossa. The dorsolateral flange is asymmetrical with a sharp lateral apex in some specimens (e.g., DMNH 2014-12-490,) as in juvenile *Edmontosaurus annectens* [38], although the flange in several specimens are rounded (e.g., DMNH 2014-12-456) as in adult Edmontosaurus annectens [38]. A faint transverse ridge is present at the level of the lateral apex of the dorsolateral flange. The transverse ridge defines the anterior margin of the quadrate glenoid, while the mesial sagittal ridge defines the medial margin of the quadrate glenoid. Posteriorly, the ventromedial margins of the anterior part of the surangular and the caudal process is widely angled (>160). The retroarticular process is slightly recurved dorsally and has a gently arcuate posteroventral margin (UAMES 21522).

### Maxillary teeth

**Fig 9:**
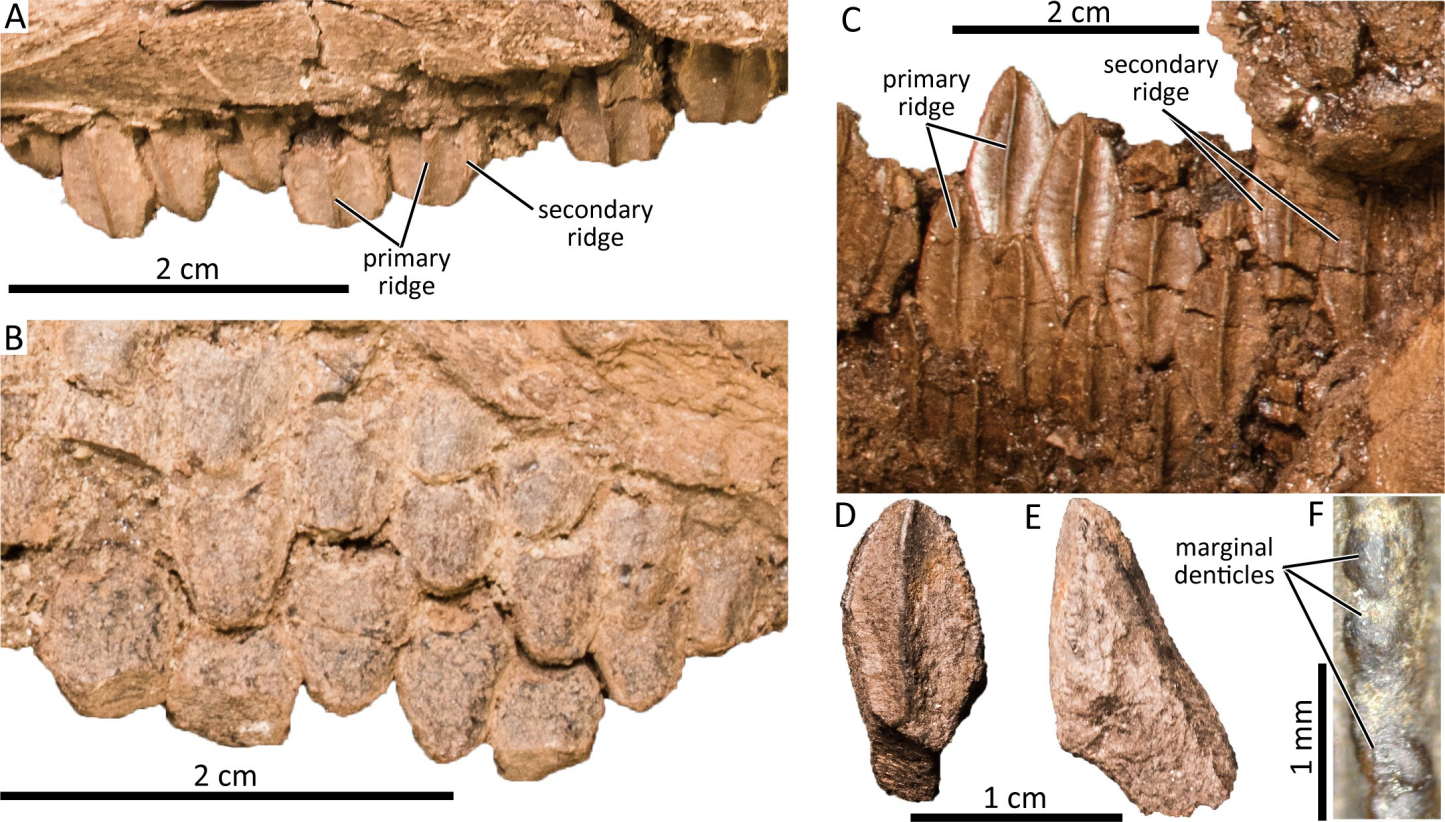
Maxillary teeth (DMNH 22718) in labial (A) and lingual (B) views. Dentary teeth (DMNH 22851) in lingual view (C). Isolated dentary tooth (DMNH 22338) in lingual (D) and mesial (E) views. Close-up of the marginal denticles of the dentary tooth (F).

The anterior half of a hadrosaurine maxilla that preserves maxillary teeth in nearly the original positions (DMNH 22718) demonstrates a maximum of two functional maxillary teeth per alveolus (Fig 9A and 9B). One prominent primary ridge and a maximum of one faint secondary ridge distal to the primary ridge exist on the labial enameled surface of the maxillary teeth. The primary ridge is positioned at the midline of the tooth crown and is nearly straight in all of the teeth. The apical half of the maxillary tooth crowns bear small marginal denticles. The dental battery houses a maximum of two replacement teeth per alveolus in addition to a functional tooth. The newer replacement teeth overlap the prior one mediodorsally.

### Dentary teeth

Left and right hadrosaurine dentaries compressed against each other (DMNH 22851) demonstrate at least two functional teeth are present in each of the dentary’s anterior tooth rows (Fig 9C–9F), although the detail on the posterior half of the dental battery is unavailable. A maximum of three teeth are present in an alveolus of the anterior region of a right dentary. The tooth crowns are diamond-shaped and the available specimens show variant height/width ratios ranging from 2.04–2.33. The tooth crowns bear one primary ridge and a secondary ridge in some teeth (mesial to the primary ridge, if present) on its lingual enameled surface as in some *Edmontosaurus regalis* (present in a few teeth of CMN 2289) and lambeosaurines [53, 75]. The primary ridge on the dentary teeth is positioned at the midline of the tooth crown and is slightly sinuous. Small marginal denticles are present on both margins of the apical half of the dentary teeth. Each denticle consists of one small knob-like protrusion (Fig 9F) as in *Edmontosaurus* [58, 73] and *Kamuysaurus japonicus* [43], but unlike those of *Gryposaurus notabilis* (AMNH FARB 8526) and *Corythosaurus casuarius* (AMNH FARB 3971) that are formed of multiple small protrusions. In an isolated teeth (DMNH 22338), the root is angled more than 140° from the tooth crown in distal/mesial aspect.

### Phylogenetic analysis

**Fig 10:**
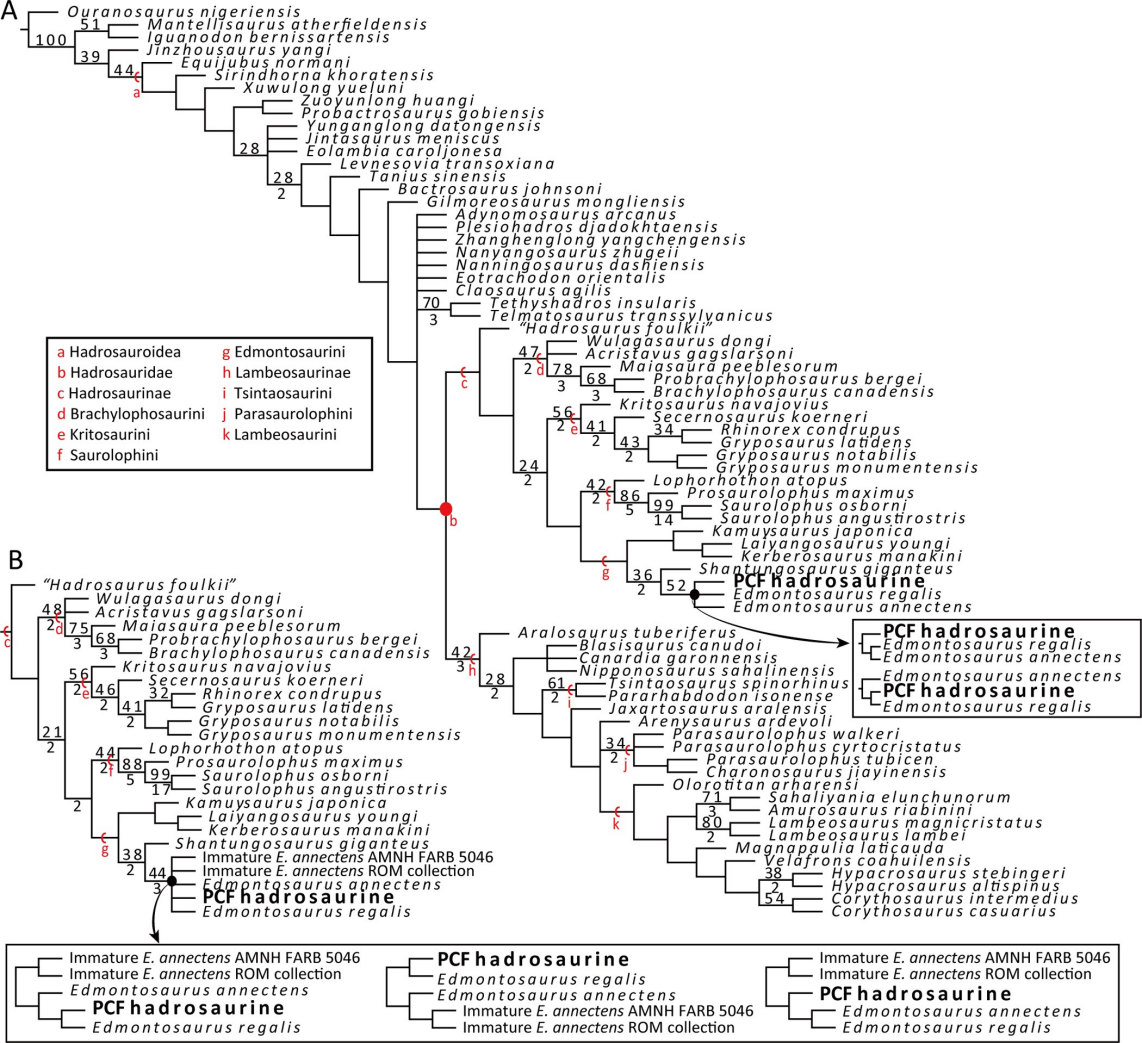
(A) Strict consensus tree of the MPTs obtained from the first phylogenetic analysis which excludes the two immature *Edmontosaurus annectens* OTUs. Numbers above each branch represent bootstrap values and those below the branch represent Bremer decay values. Bootstrap values below 20 and Bremer decay values below 1 are not shown. The polytomy consisting of the PCF hadrosaurine + *Edmontosaurus annectens* + *Edmontosaurus regalis* is the result of the two conflicting topologies shown in the small box. (B) A simplified strict consensus tree of MPTs obtained from the second phylogenetic analysis which incorporated two juvenile *Edmontosaurus annectens* OTUs, showing relationship within Hadrosaurinae. The polytomy consisting of the PCF hadrosaurine + *Edmontosaurus annectens* (including immature OTUs) + *Edmontosaurus regalis* is the result of the three conflicting topologies shown in the bottom box.

Two phylogenetic analyses were conducted in this study (see Materials and Methods). The first analysis resulted in 384 equally most parsimonious trees (MPTs) of 1148 steps, each with a consistency index of 0.441 and a retention index of 0.844. The topology of the strict consensus tree is similar to that of Kobayashi et al. [43] other than the inclusion of the PCF hadrosaurine (Fig 10A). The analysis recovers the PCF hadrosaurine within an unresolved polytomy with *Edmontosaurus annectens* and *Edmontosaurus regalis*.

Among the four synapomorphies recovered for Edmontosaurini, the PCF hadrosaurine shares the marginal denticle of dentary teeth composed of one rounded knob (12–1), the thick lip-like structure of the premaxillary oral rim (74–4), and the absence of the specialized development of the nasal (163–0). The PCF hadrosaurine exhibits two of the six synapomorphies that define the clade consisting of *Shantungosaurus giganteus* + *Edmontosaurus*: a finger-shaped anterior process of the parietal (204–1), and a weakly deflected occipital condyle (234–1). Although the PCF hadrosaurine does not share three synapomorphies of the clade (number of dental alveoli more than 45 (1–2), jugal process of the postorbital wide anteroposteriorly (181–2), and short squamosal process of the postorbital (183–1)) which are ontogenetically variable [42], the first analysis infers that these features may appear in mature individuals of the PCF hadrosaurine. The PCF hadrosaurine and *Edmontosaurus* share a reduced postorbital process of the laterosphenoid (221–1). Among the other six synapomorphies of the unresolved PCF hadrosaurine and *Edmontosaurus* clade, the analysis indicates that the short edentulous slope of the dentary (38–0), the moderate anterior inclination of the coronoid process (48–1), the absence of the deep pocket of the postorbital (182–0) of the PCF hadrosaurines are all characters that are ontogenetically variable in their expression. Their presence or absence cannot be objectively assessed or considered phylogenetically informative in the absence of equivalent ontogenetic exemplars of each of the pertinent taxa.

The first analysis recovered a combination of three characters potentially unique to the PCF hadrosaurine: dentary teeth with a secondary ridge (6–2), basisphenoid without a ventral transverse ridge (217–1), and an anteroposteriorly elongated maxilla (110–0). The presence of a secondary ridge on the dentary teeth is shared with lambeosaurines and also with some teeth of *Edmontosaurus regalis* (CMN 2289). The absence of the ventral transverse ridge could be intraspecific variation since expression of this structure is also present in *Edmontosaurus annectens* (ROM 59786). Alternatively, since a lambeosaurine is now recognized in the Prince Creek Formation [10], the feature shared with derived lambeosaurines *Corythosaurus* and *Hypacrosaurus* may simply indicate that this element belongs to a member of Lambeosaurinae, although it also has a hadrosaurine feature (see description section). The anteroposteriorly long maxilla is unique to the PCF hadrosaurine and *Laiyangosaurus youngi* [51].

The polytomy comprising the PCF hadrosaurine + *Edmontosaurus* in the strict consensus tree is a result of two conflicting topologies recovered in the 384 MPTs. In one topology, the PCF hadrosaurine is positioned as the sister taxon to the *Edmontosaurus* clade (*Edmontosaurus annectens* + *Edmontosaurus regalis*) because the two species of *Edmontosaurus* are united by a single character to the exclusion of the PCF taxon: a straight primary ridge on the dentary tooth crown (8–0; slightly curved or sinuous in the PCF specimens). In the second recovered topology, the PCF hadrosaurine is the sister-taxon to *Edmontosaurus regalis* to the exclusion of *Edmontosaurus annectens*. The PCF hadrosaurine and *Edmontosaurus regalis* are united in this scenario by the shared presence of a horizontal shelf on the postorbital process of the jugal (354–1).

The second cladistic analysis which incorporated two immature *Edmontosaurus annectens* as separate OTUs recovered 576 MPTs of 1154 steps (CI = 0.438, RI = 0.842). The strict consensus tree recovered the PCF hadrosaurine within the polytomy of *Edmontosaurus* OUTs (Fig 10B). The polytomy is a result of three conflicting topologies (Fig 10B, bottom box). PCF hadrosaurine is monophyletic with *Edmontosaurus regalis* in two topologies, supported by the anteroposteriorly short prenarial region of the premaxilla (353–0). Alternatively, the other topology positions PCF hadrosaurine as a sister taxon of the clade of adult *Edmontosaurus annectens* OTU + *Edmontosaurus regalis*, the clade supported by a straight primary ridge on the dentary tooth crown (8–0). It is important to note that the immature *Edmontosaurus annectens* OTUs and mature *Edmontosaurus annectens* OTU did not form a monophyletic clade with each other to the exclusion of *Edmontosaurus regalis* and the PCF hadrosaurine in two of the three topologies recovered.

## Discussion

### Taxonomic status of “*Ugrunaaluk kuukpikensis*”

Mori et al [36] proposed eight diagnostic characters for *Ugrunaaluk kuukpikensis*. Among the eight characters, three were proposed to differentiate *Ugrunaaluk kuukpikensis* from *Edmontosaurus*, four from *Edmontosaurus annectens*, and one from *Edmontosaurus regalis*. However, the three characters proposed to differentiate *Ugrunaaluk* from *Edmontosaurus* are problematic. The first character, a posterolaterally projected circumnarial ridge without a premaxillary vestibular promontory, is present in at least one immature *Edmontosaurus annectens* specimen (ROM 53525). The second, an anterodorsally inclined posterior margin of the anterior process of the jugal, is also present in immature specimens of *Edmontosaurus annectens* (e.g., DMNH EPV.95220) and the degree of inclination is variable intraspecifically in large, assumedly mature specimens as well (Fig 4C–4D). While the third character, absence of the deep postorbital fossa, is markedly different from immature specimens of *Edmontosaurus annectens*, the character cannot be legitimately assessed in *Edmontosaurus regalis* because no specimens of equivalent size and ontogenetic stage as the PCF specimens have been reported for *Edmontosaurus regalis*. Therefore, the wide and shallow postorbital fossa in the Arctic PCF hadrosaurine that Mori et al. [36] considered diagnostic could potentially be present in immature individuals of *Edmontosaurus regalis*. This is pertinent because the postorbital fossa in large *Edmontosaurus regalis* specimens is markedly wider than that of comparably sized *Edmontosaurus annectens*. Based on these shortcomings, the characters Mori et al. [36] proposed are insufficient to diagnose and differentiate *Ugrunaaluk* from *Edmontosaurus*. Additionally, all of the eight characters are widely seen among members of Hadrosaurinae, and are thus inappropriate to diagnose *Ugrunaaluk* as a distinct taxon.

The unresolved clade of *Edmontosaurus* + the PCF hadrosaurine recovered in the present phylogenetic analyses (Fig 10) indicate that the PCF hadrosaurine should be considered a member of the *Edmontosaurus* clade. The phylogenetic patterns within the unresolved polytomies further provide additional support for deeply nesting the PCF hadrosaurine within the *Edmontosaurus* clade, potentially more closely related to *Edmontosaurus regalis* than *Edmontosaurus annectens* (Fig 10B). In fact, one of the synapomorphies of the PCF hadrosaurine + *Edmontosaurus* clade (presence of a short postorbital process of the laterosphenoid) is one of the diagnostic characters of *Edmontosaurus* [35, 37]. Given the relationships recovered here that show the PCF hadrosaurine is a member of the *Edmontosaurus* clade, the absence of any unequivocally diagnostic characters differentiating the PCF hadrosaurine from *Edmontosaurus*, and the presence of at least one diagnostic character of *Edmontosaurus* in the PCF hadrosaurine, the present work assigns the PCF hadrosaurine to *Edmontosaurus*, and the name “*Ugrunaaluk*” is considered a junior synonym of *Edmontosaurus*.

At the same time, the PCF *Edmontosaurus* exhibits some differences from *Edmontosaurus annectens* and *Edmontosaurus regalis* as previously documented [36–38]. The most significant difference is the relatively anteroposteriorly elongated maxilla in the PCF *Edmontosaurus*. The maxilla is expected to elongate through ontogeny along with overall cranial elongation [30, 56, 57], and the height-length ratio of the maxilla in Edmontosaurini shows negative allometry (Fig 2C), suggesting that the elongated maxilla of the PCF *Edmontosaurus* is not a result of the relative immaturity of the specimens feature. Taxonomic significance of other features that may be different from *Edmontosaurus annectens* and *Edmontosaurus regalis* is ambiguous. The anteroposteriorly-elongated lacrimal in the small Alaskan specimens differs from that of adult *Edmontosaurus regalis*, but this could be a size and ontogeny-dependent feature and the lack of ontogenetically-equivalent specimens of *Edmontosaurus regalis* makes this hypothesis currently untestable. The large lateral exposure of the quadratojugal suggested by Mori et al. [36] may also differ from the condition in *E*. *regalis*, but it is important to note that quadratojugal morphology is known to be variable intraspecifically (see description), thus it seems unlikely to be a taxonomically valid character. The absence of the transverse ridge between basipterygoid processes of the basisphenoid and the presence of the secondary ridge of the dentary teeth are recovered as unique combination to the PCF *Edmontosaurus* in the present phylogenetic analyses, but taxonomic importance of these characters are questionable due to their intraspecific variations (see description) and the multitaxic nature of PCF hadrosaurids [10].

Therefore, the elongated maxilla of the PCF *Edmontosaurus* is the only feature that may be reasonably different from the other members of *Edmontosaurus* (Table 1). However, this feature might prove inadequate to diagnose the taxon since an anteroposteriorly-elongated maxilla is also present in another member of the Edmontosaurini, *Laiyangosaurus youngi* [51]. For the reasons listed above, the validity of the species “*kuukpikensis”* is left as questionable and unresolved. Therefore, we agree with Gangloff and Fiorillo [32], Mori et al. [36], as well as the subsequent works by Xing et al. [37] and Wosik et al. [38] that adult material is critical for determining the finer-scale taxonomy of this hadrosaur. We advocate for a more conservative approach and recommend the PCF hadrosaur be referred to *Edmontosaurus* sp. until further discoveries of more mature individuals from the Prince Creek Formation and/or comparably immature *Edmontosaurus annectens* and *Edmontosaurus regalis* allow comparison of anatomy of individuals of all three taxa from the same ontogenetic stages.

**Table 1:** Comparisons among *Edmontosaurus*.

		PCF *Edmontosaurus*	*E*. *annectens*	*E*. *regalis*	Ontogenetic variation	Intraspecific variation	Lambeosauine possibility	Taxonomic validity
**Character potentially differentiating the PCF *Edmontosaurus* from *E*. *annectens* and *E*. *regalis***								
	Height/length ratio of maxilla	<0.34	>0.37	0.39	Ratio get smaller	Present	Absent	Possible
**Characters potentially differentiating the PCF *Edmontosaurus* from *E*. *annectens* and *E*. *regalis*, but cannot be tested**								
	Lacrimal shape	Anteroposteriorly elongated, wedge-shaped	Robust, blunt anterior end	Robust, blunt anterior end	?	?	Unlikely	Dubious
	Bi-lobed ventral process of the predentary	Short	Long	Long	?	?	?	Dubious
	Ventral transverse ridge between the basipterygoid processes of the basisphenoid	Absent	Present	Present	?	Present	Possible	Dubious
	Secondary ridge of dentary teeth	Present	Absent	Absent	?	Present	Absent	Dubious

### Influence of latitudinal habitat difference on hadrosaurine evolution

The reattribution of the PCF hadrosaurine to *Edmontosaurus* highlights a seemingly great latitudinal distribution for genus *Edmontosaurus*: ranging from northern Colorado (approximately 41° N, present; *Edmontosaurus annectens*) [76, 77] to northernmost Alaska (approximately 70° N, present; PCF *Edmontosaurus* sp.), and *Edmontosaurus regalis* in between the two regions (present-day western Canada). However, in the modern ecosystems there are at least two genera of mammalian vertebrates that have ranges of comparable latitudinal magnitude, the wolf (*Canis lupus*) and the wild sheep of North America (*Ovis* sp.). Further, the cougar (*Puma concolor*) has arguably the widest range of any terrestrial mammal in the Western Hemisphere, ranging from northern Canada to extreme southern South America. Thus the latitudinal range seen in the fossil record for *Edmontosaurus* is not unprecedented, though deserved of elaboration.

**Fig 11:**
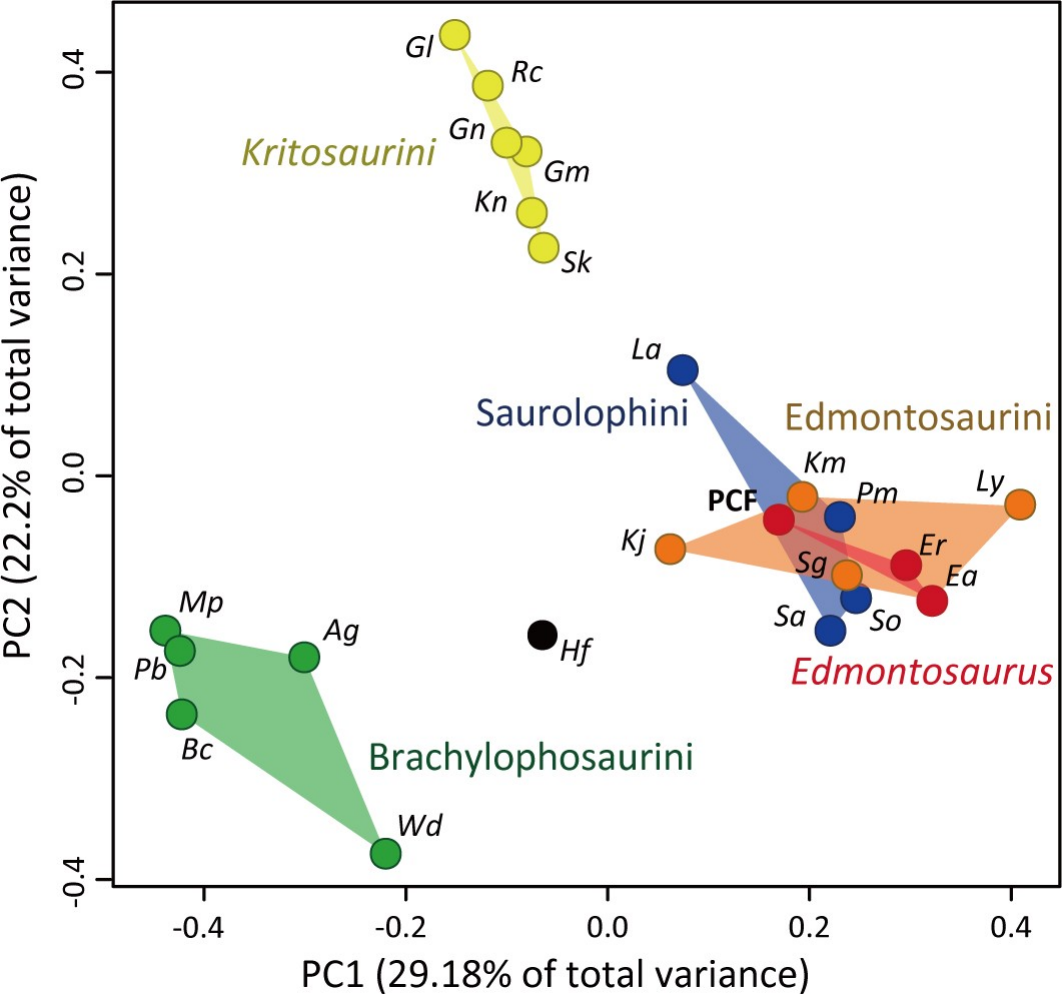
Morphospace of hadrosaurines recovered from the principal coordinate analysis using the phylogenetic cranial characters. Abbreviations: *Ag*: *Acristavus gagslarsoni*, *Bc*: *Brachylophosaurus canadensis*, *Ea*: *Edmontosaurus annectens*, *Er*: *Edmontosaurus regalis*, *Gl*: *Gryposaurus latidens*, *Gm*: *Gryposaurus monumentensis*, *Gn*: *Gryposaurus notabilis*, *Hf*: *Hadrosaurus foulkii*, *Kj*: *Kamuysaurus japonicus*, *Km*: *Kerberosaurus manakini*, *Kn*: *Kritosaurus navajovius*, *La*: *Lophorhothon atopus*, *Ly*: *Laiyangosaurus youngi*, *Mp*: *Maiasaura peeblesorum*, *Pb*: *Probrachylophosaurus bergei*, *Pm*: *Prosaurolophus maximus*, *Rc*: *Rhinorex condrupus*, *Sa*: *Saurolophus angustirostris*, *Sg*: *Shantungosaurus giganteus*, *Sk*: *Secernosaurus koerneri*, *So*: *Saurolophus osborni*, *Wd*: *Wulagasaurus dongi*.

The significant morphological similarities within the genus *Edmontosaurus* are also demonstrated with the small phylogenetic morphospace (Fig 11) and the morphological partial disparity (Table 2). The partial disparity/OTU of *Edmontosaurus* (3.40%) is lower than the hadrosaurine average (4.41%), suggesting that the latitudinal habitat differences did not increase morphological disparity of *Edmontosaurus*. *Gryposaurus*, a genus with moderate latitudinal distribution from southern Utah (approximately 38° N, present) [78] to southern Alberta (approximately 51° N, present) [79], and *Saurolophus*, a genus which is expected to have a “ghost distribution” in the Arctic [80], have only moderate partial disparity/OTU (4.50% and 4.79%, respectively). It should be noted that genus-level comparisons among fossil taxa must be treated carefully, as genera do not represent the same meaningful biological units under the concept of biological species [81], and that the “latitudinal ranges” of *Edmontosaurus*, *Gryposaurus*, and *Saurolophus* are formed based on non-contemporaneous species. Although these possible artefacts must be treated with special cautions, the low partial disparity/OTU of *Edmontosaurus*, *Gryposaurus*, and *Saurolophus* may suggest that overall hadrosaurine morphological disparity is not strongly affected by their latitudinal geographic distribution. Small morphological disparities despite large latitudinal distributions may be related to the relatively low latitudinal temperature gradient during the latest Cretaceous compared to the present day [82], which might not require much morphological adaptations for latitudinal distributions.

**Table 2:** Partial disparities of hadrosaurines.

	Genus	PD/Genus	PD/Genus (%)	PD/OTU (%)
Edmontosaurini	*Edmontosaurus*	0.0224	10.21	3.40
	*Kamuysaurus*	0.0102	4.64	4.64
	*Kerberosaurus*	0.0050	2.28	2.28
	*Laiyangosaurus*	0.0126	5.76	5.76
	*Shantungosaurus*	0.0086	3.93	3.93
Kritosaurini	*Kritosaurus*	0.0077	3.50	3.50
	*Gryposaurus*	0.0296	13.50	4.50
	*Rhinorex*	0.0095	4.35	4.35
	*Secernosaurus*	0.0059	2.71	2.71
Saurolophini	*Saurolophus*	0.0210	9.56	4.78
	*Lophorhothon*	0.0062	2.84	2.84
	*Prosaurolophus*	0.0063	2.85	2.85
Brachylophosaurini	*Acristavus*	0.0097	4.43	4.43
	*Brachylophosaurus*	0.0129	5.88	5.88
	*Maiasaura*	0.0131	5.97	5.97
	*Probrachylophosaurus*	0.0138	6.28	6.28
	*Wulagasaurus*	0.0147	6.72	6.72
	*Hadrosaurus*	0.0101	4.60	4.60

## Supporting information

S1 Data Character-taxon matrix in nexus format. (NEX)

S1 Text Character list for the phylogenetic analyses. (DOCX)

S1 Table List of ROM *Edmontosaurus annectens* used as an OTU. (XLSX)

S2 Table Hadrosaurine maxilla measurements. (XLSX)
